# Biofilm Formation Protects *Salmonella* from the Antibiotic Ciprofloxacin *In Vitro* and *In Vivo* in the Mouse Model of chronic Carriage

**DOI:** 10.1038/s41598-017-18516-2

**Published:** 2018-01-09

**Authors:** Juan F. González, Halley Alberts, Joel Lee, Lauren Doolittle, John S. Gunn

**Affiliations:** 1 Department of Microbial Infection and Immunity, Infectious Diseases Institute, The Ohio State University, Columbus, Ohio USA; 2 School of Mathematics and Natural Sciences, University of Rio Grande, P.O. Box 500 Rio Grande, OH USA

## Abstract

Typhoid fever is caused by the human-restricted pathogen *Salmonella enterica* sv. Typhi. Approximately 5% of people that resolve the disease become chronic carriers, with the gallbladder as the main reservoir of the bacteria. Of these, about 90% present with gallstones, on which *Salmonella* form biofilms. Because *S*. Typhi is a human-restricted pathogen, these carriers are the main source of dissemination of the disease; unfortunately, antibiotic treatment has shown to be an ineffective therapy. This is believed to be caused by the inherent antibiotic resistance conferred by *Salmonella* biofilms growing on gallstones. The gallstone mouse model with *S*. Typhimurium has proven to be an excellent surrogate for *S*. Typhi chronic infection. In this study, we test the hypothesis that the biofilm state confers *Salmonella* with the increased resistance to antibiotics observed in cases of chronic carriage. We found that, in the biofilm state, *Salmonella* is significantly more resistant to ciprofloxacin, a common antibiotic used for the treatment of *Salmonella*, both *in vitro* (p < 0.001 for both *S*. Typhi and *S*. Typhimurium with respect to planktonic cells) and *in vivo* (p = 0.0035 with respect to control mice).

## Introduction

Typhoid fever is primarily caused by the human-restricted pathogen *Salmonella enterica* sv. Typhi (*S*. Typhi). This disease is a global problem that affects millions of people and causes over 600,000 deaths annually^1^. Typhoid fever is an acute illness with symptoms that include high fever, malaise, and abdominal pain. This disease is of special importance in developing nations where the lack of clean water and poor sanitation favor the capacity of the bacteria to spread. Although the incidence of Typhoid in developed countries is low, travelers are still at risk^2^.

Typhoid has a mortality rate of 2–3% even with adequate antibiotic therapy. The emergence of multidrug-resistant (MDR) strains of *S*. Typhi is a significant problem as inexpensive and readily available antibiotics including ampicillin, chloramphenicol, trimethoprim-sulfamethoxazole and streptomycin are frequently ineffective^3,4^. Although resistance to ciprofloxacin, a second-generation fluoroquinolone, is increasing, it is still recommended as first line therapy for children and adults^5–7^.

In most cases, the infection is resolved and the patient recovers, but approximately 5% of people infected with *S*. Typhi fail to clear the bacteria within one year and become chronic carriers. Carriers are typically asymptomatic and can spread the disease through fecal shedding. The chronic carrier state is associated with *Salmonella* colonization of the biliary tract and is positively correlated with the presence of gallstones (GS); in fact, approximately 90% of chronically infected carriers have GS^8^. Antibiotic treatment is ineffective in the resolution of chronic *S*. Typhi colonization, making gallbladder removal the only effective therapy^2,9–11^.

The presence of *Salmonella* biofilms on the gallstones of both typhoid carrier patients and mice (in a gallstone model of chronic carriage) has been previously demonstrated^12,13^. Biofilms are bacterial communities that attach to a biological or non-biological surface and are enveloped by a bacterial-initiated matrix. This structure allows bacteria to survive in hostile conditions such as exposure to UV light, metal toxicity, acid exposure, dehydration and salinity, phagocytes, and several antibiotics and antimicrobial agents^14^. It is hypothesized that this growth state accounts for the recalcitrance *S*. Typhi shows to antibiotic treatment in chronic carriers. Since the host range of *S*. Typhi is restricted to humans, *Salmonella enteriva* sv. Typhimurium (*S*. Typhimurium) infection of mice has been widely used to study typhoid fever pathogenesis and immunity. The gallstone mouse model developed by us using *S*. Typhimurium has proven to be a powerful tool that mimics human chronic carriage^12^.

In this study, we test the hypothesis that the biofilm state confers to both *S*. Typhimurium and *S*. Typhi substantial resistance to the antibiotic ciprofloxacin compared to planktonic cells. Experiments *in vitro* assess the hypothesis by colony-forming unit (CFU) quantification and confocal microscopy, while our gallstone mouse model was utilized *in vivo* to examine if biofilms on gallstones conferred *S*. Typhimurium with resistance to ciprofloxacin.

## Results

It is widely accepted that bacterial biofilms are more resistant to environmental stress, including the presence of antimicrobial agents, than free-swimming bacteria. In order to investigate whether biofilm formation on gallstones contributes to antibiotic resistance typically observed in *S*. Typhi human chronic carriers treated with antibiotics, we conducted a series of experiments comparing the survival of bacterial cultures in both planktonic and biofilm states exposed to the antibiotic ciprofloxacin. Such experiments were performed both *in vitro* and in a murine gallstone model. *In vitro* assays utilized cholesterol-coating of the wells to mimic gallstones^12^.

### *In vitro* antibiotic susceptibility

**Figure 1:**
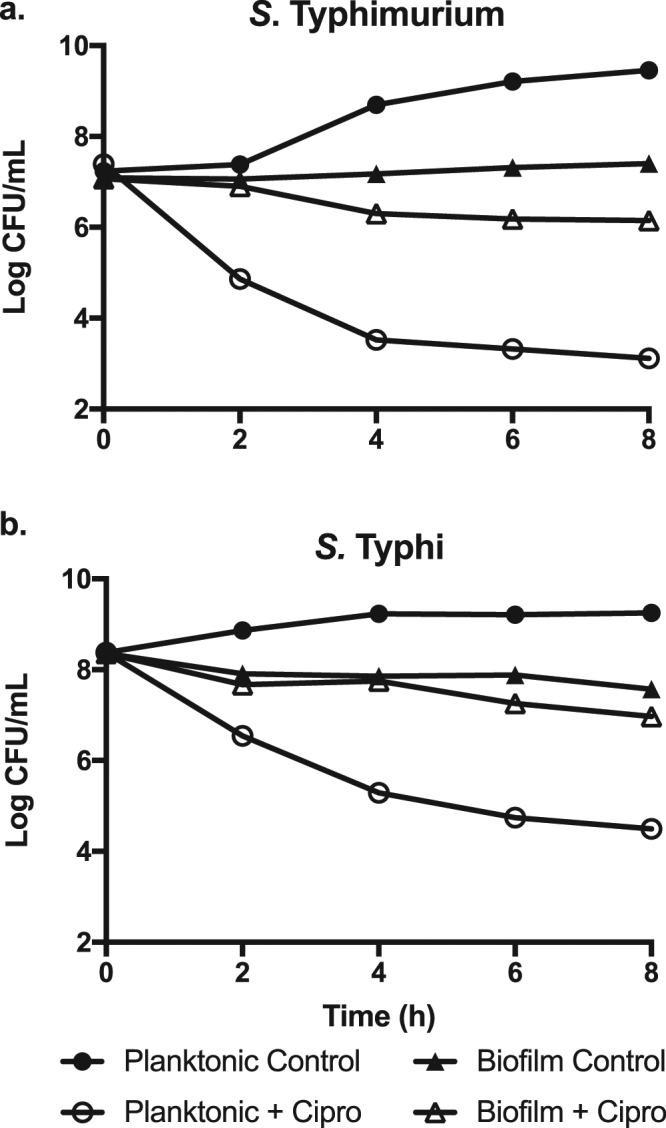
The *in vitro* effect of ciprofloxacin on planktonic vs. biofilm *Salmonella* cultures. (**a**) *S*. Typhimurium cultures were treated with 0.125 μg/mL ciprofloxacin and (**b**) *S*. Typhi with 0.02 μg/mL for a total of 8 hours. CFUs were enumerated every 2 hours. Data is presented as mean and SD. There are significant differences between ciprofloxacin treated and untreated planktonic cultures of both *S*. Typhimurium (*P* < 0.001) and *S*. Typhi (*P* < 0.001). Comparisons of data were performed using one-way analysis of variance (ANOVA) followed by Tukey’s Studentized range test at the 8 hr time point. For all parameters *P* < 0.05 was considered the level of significance. The data presented are representative of three independent experiments each performed in triplicate.

The The Minimum Inhibitory Concentration (MIC) for ciprofloxacin was determined to be 0.125 µg/mL for JSG210 (*S*. Typhimurium) and 0.5 µg/mL for JSG624 (*S*. Typhi) which puts our two laboratory strains in the “intermediately susceptible” range for *Salmonella* (data not shown)^15,16^. These concentrations were used as a baseline for further broth and biofilm culture analyses. To compare the effect of ciprofloxacin on the different growth conditions, planktonic cell numbers were normalized to the CFU in 5-day biofilm cultures and the effect of the antibiotic was monitored for 8 hours (see Methods section). The antibiotic concentration used against *S*. Typhimurium was the same as the MIC, while for *S*. Typhi the concentration was decreased to 0.02 µL/mg after determining that the MIC calculated for *S*. Typhi using the dilution method in microtiter plates was too high for growth in tubes on a rotator drum. A dramatic and steady decrease in CFU numbers over time was observed for *S*. Typhimurium planktonic cultures, reaching a 6-log difference after 8 hours (p < 0.001), with respect to the control (Fig. 1a). In contrast, ciprofloxacin treatment had a modest one-log difference with respect to biofilm cell numbers. *S*. Typhi showed a similar trend over the 8 hours tested (Fig. 1b), dropping steadily until reaching an almost 5-log decrease after 8 hours (p < 0.001) for planktonic cultures, while biofilms were reduced less than one log. In conclusion, in an *in vitro* model mimicking *in vivo* chronic carriage conditions, *Salmonella* biofilms were dramatically more resistant to killing by ciprofloxacin.

### Biofilm integrity

**Figure 2:**
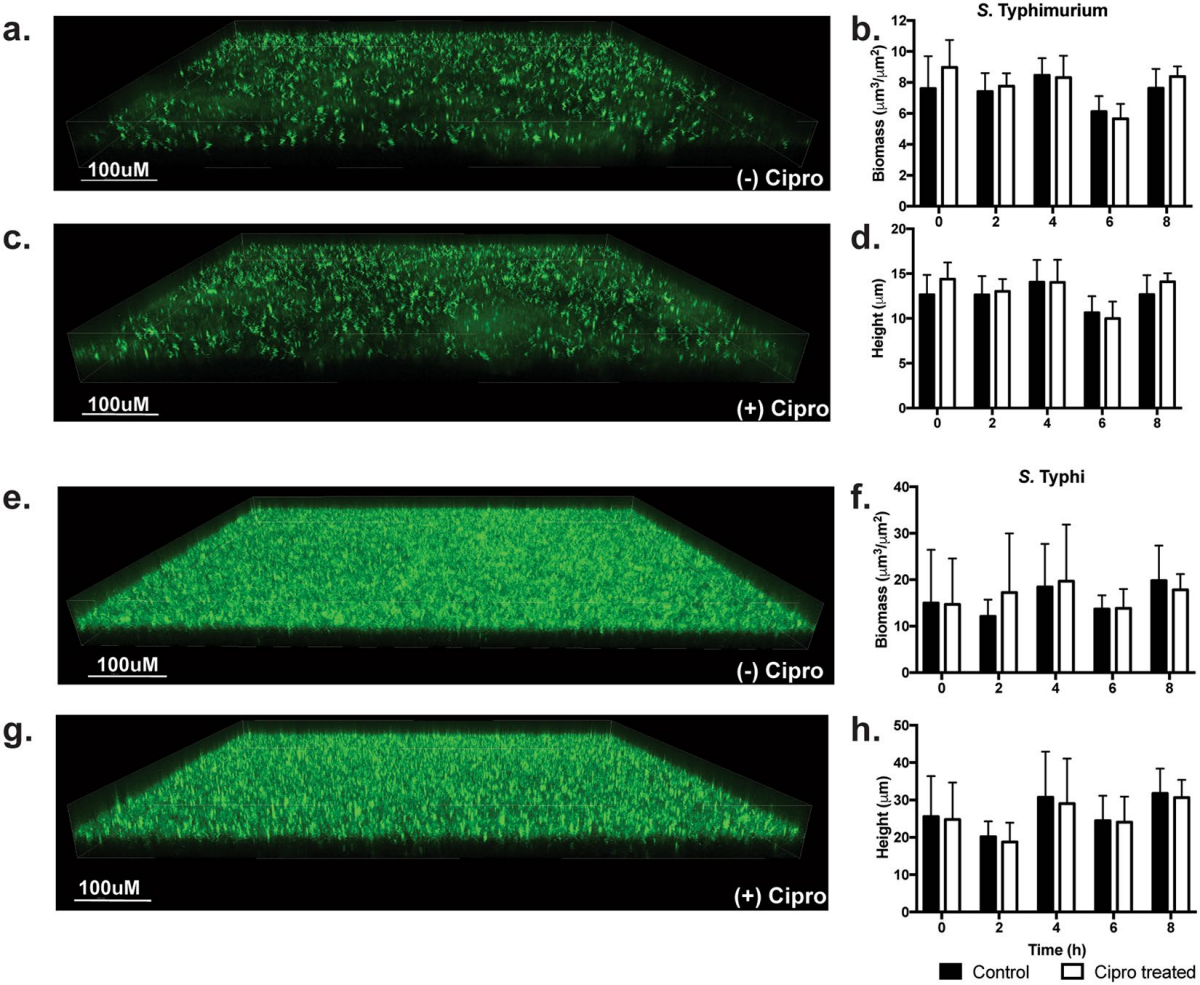
Effect of ciprofloxacin on biofilm integrity *in vitro*. Representative images of 5-day biofilms of *S*. Typhimurium control (untreated) (**a**), ciprofloxacin-treated for 8 h just prior to imaging (**c**) and *S*. Typhi control (untreated) (**e**), ciprofloxacin-treated for 8 h just prior to imaging (**g**). Experimental conditions were the same as for CFU enumeration: microtiter plates were fixed in 2% PFA every two hours and saved for later imaging (5 time points total). Data is presented as mean and SD. Biomass and average biofilm height were calculated with the software package COMSTAT and comparisons made between control- and ciprofloxacin-treated *S*. Typhimurium (**b** and **d**) and *S*. Typhi (**f** and **h**). No significant differences were observed in any parameter.

In order to evaluate the integrity of *Salmonella* biofilms after treatment with ciprofloxacin (same conditions as CFU enumeration), GFP-labeled biofilms were observed under an inverted confocal microscope, which gives an indication of biofilm architecture and biomass. As can be seen in Fig. 2, no visual difference was observed between the biofilm cultures grown in growth media alone or media supplemented with ciprofloxacin for 8 hours prior to imaging for either *S*. Typhimurium (Fig. 2a and c) or *S*. Typhi (Fig. 2e and g). Additionally, no significant difference was found in either biomass or average height in the biofilms calculated from Z-stacks for either serovar (Fig. 2b and d,f and h). The clear differences in biofilm formation between *S*. Typhimurium and *S*. Typhi are likley caused by the need for different growth conditions (see Methods section) due to the inability of *S*. Typhi to form robust biofilms in 96-well plates.

### *In vivo* antibiotic susceptibility

**Figure 3:**
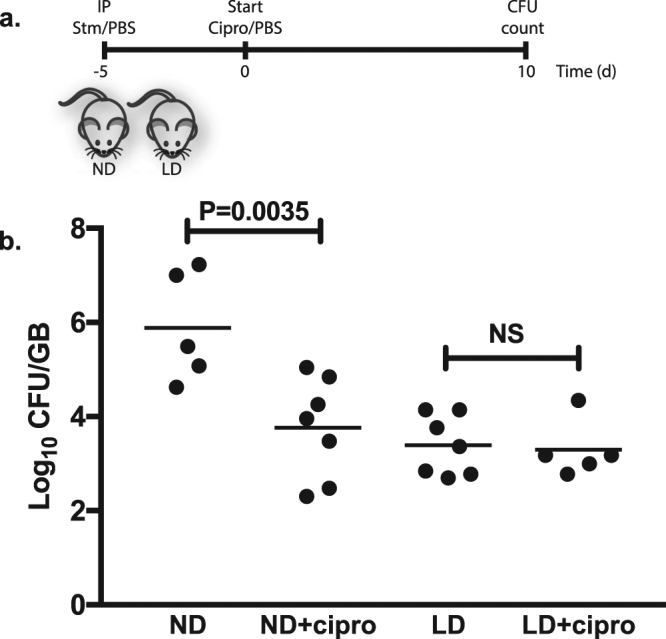
The *in vivo* effects of ciprofloxacin on planktonic vs. gallstone biofilms. (**a**) Experimental setup: mice fed for 8 weeks with a ND or LD received PBS or 5 × 10^4^ CFU *S*. Typhimurium. After 5 days (to allow biofilm development for LD mice), a regimen of ciprofloxacin treatment (or PBS control) was initiated and continued for 10 days. (**b**) Bacterial loads in mice GBs. The limit of detection was 10 CFU/ml. Bars represent the mean. Comparisons of data were performed using one-way analysis of variance (ANOVA) followed by Tukey’s Studentized Range test. For all parameters, *P* < 0.05 was considered significant. ND = normal diet; LD = lithogenic diet; IP = intraperitoneal; GB = gallbladder.

We also compared the effects of ciprofloxacin *in vivo* using a murine gallstone model^12^. In this model, mice are fed a lithogenic diet (LD) for 6 to 8 weeks, which consistently caused gallstone development. *Salmonella* uses this scaffold on which to form a biofilm in humans and mice^12^. Mice on a normal diet (ND) do not develop gallstones; consequently *Salmonella* does not have a substrate on which to develop a biofilm. Five days after infection, ciprofloxacin treatment was initiated and continued daily for 10 days (1 mg/kg/day). At the end of the treatment, the mice were sacrificed to examine bacterial numbers in the gallbladder. The *in vivo* results parallel the *in vitro* results (Fig. 3), as mice on a ND showed a high bacterial gallbladder load of around 1 × 10^6^ CFU/mL and presented a significant 2-log reduction (p = 0.0035) when treated with ciprofloxacin. In contrast, mice fed a LD reached bacterial loads in the gallbladder of almost 1 × 10^4^, which remained unchanged after antibiotic treatment. LD-fed mice infected under identical conditions demonstrated copious biofilm development on gallstone surfaces^12,17,18^. Thus, these data suggest that the biofilm lifestyle in the gallbladder protects bacteria from antibiotic clearance.

## Discussion

Third generation fluoroquinolones have replaced first-line drugs to become standard for the treatment of acute Typhoid fever with some success observed in chronic carriers^19–22^. However, with ever increasing antibiotic resistance coupled with the lack of consistent success of antibiotics in chronic carriers, antibiotics alone are usually not sufficient and cholecystectomy is necessary to fully clear the gallbladder infection^23^. This is believed to be caused by *Salmonella* biofilm formation on gallstones, as biofilms have been demonstrated to provide bacteria recalcitrance towards antibiotics. In this study, we test this hypothesis *in vitro* and *in vivo*.

Here, we demonstrated that *Salmonella* biofilms are much more resistant to ciprofloxacin, an antibiotic of the fluoroquinolone family widely used to control *Salmonella* infections^24^. *In vitro*, both *S*. Typhimurium and *S*. Typhi biofilms show a decrease in CFU numbers of less than 1-log after 8 hours of antibiotic treatment. Conversely, planktonic cells show a 5–6 log reduction. Additionally, no differences in biofilm structure, biomass or average height were detected after antibiotic treatment (Fig. 2). It is worth mentioning that an observable difference is evident between *S*. Typhimurium and *S*. Typhi biofilms (Fig. 2a and c vs. e and g). This is due to their different growth conditions: *S*. Typhimurium biofilms were grown in 96-well plates while *S*. Typhi were grown in 8-well chamber slides, the latter resulting in a more robust biofilm formation. For *in vivo* assessment, we used the gallstone mouse model, which mimics human gallbladder carriage, as most human carriers possess gallstones^25^. *S*. Typhimurium is used in this model, as *S*. Typhi is human-specific, consequently resulting in a non-productive infection in mice. *S*. Typhimurium showed no reduction in CFU numbers after 10 days of ciprofloxacin treatment in our murine gallstone model, while dropping approximately 2-logs in mice without gallstones. These results are consistent with the hypothesis that the presence of gallstones accentuates *Salmonella* resistance towards antibiotics.

Biofilms have been shown to provide bacteria with protection against antimicrobial agents in various environments including chronic infections associated with cystic fibrosis, infective endocarditis and chronic otitis media^26^. Furthermore, biofilm resistance to ciprofloxacin has been shown for various organisms including *P*. *aeruginosa*, where this antibiotic reduced CFU numbers in urinary catheters by only two-logs^27^. Additionally, *Burkholderia cepacia* biofilms were found to be 150 times more resistant to ciprofloxacin than planktonic cells^28^ and *in vitro* evaluation of *Klebsiella pnuemoniae* biofilm resistance to ciprofloxacin yielded a modest one log reduction in CFUs^29^. Finally, *Proteus mirabilis* biofilms showed a small 0.1-log reduction in CFUs *in vitro* after ciprofloxacin treatment^30^. Regarding *Salmonella*, high levels of *S*. Typhimurium biofilm resistance to ciprofloxacin *in vitro* have been previously reported, as a high dose of 1000 μg/mL for one hour produced only a modest 0.7 log reduction in viable cell numbers in biofilms while the ciprofloxacin MIC for planktonic cells was 0.125 μg/mL^31^. While we used a much lower dose of 0.125 μg/mL for our biofilms, our results are consistent with these as we only observed a decrease in CFU numbers of less than 1 log, even after 8 hours of treatment (Fig. 1a). Clinical isolates have also been shown to have an increased resistance towards ciprofloxacin when grown in biofilms. A study of 194 clinical isolates from 13 different serotypes in Greece showed that 56% were biofilm-forming and, although they showed a modest 2.8% increased resistance towards ciprofloxacin, the minimum inhibitory concentration for bacterial growth (MICBR_50_) increased from <0.25 to 1 mg/L^32^. An *in vitro* study of 30 clinical isolates of *S*. Typhi in Pakistan, showed that 23 were robust biofilm producers. When these were exposed to a dose of 1 μg/mL of ciprofloxacin, planktonic cultures were completely eliminated, while biofilms were reduced from 10^4^ to 10^1^ but never eradicated^33^. Contrary to our data, ciprofloxacin was shown in one study to significantly lower the biofilm production of *S*. Typhimurium, albeit under different conditions than those used in our study. Majtan and colleagues found that the content of exopolysaccharides (EPS) decreased in several clinical isolates after a 24 hour incubation in sub-inhibitory concentrations of ciprofloxacin^34^. Their data originate from a study focused on the effects of sub-inhibitory concentrations of different antibiotics acting as signal molecules on biofilm formation, while our experiments were performed on fully formed biofilms. Additionally, their clinical isolates had lower MICs than our strains, which had an intermediate susceptibility, and they measured EPS as a gauge of biofilm content, while our study focused on CFU counts.

Our data shows that *Salmonella* biofilms on GSs/cholesterol-coated surfaces are much more resistant to the primary antibiotic used clinically against human infections, ciprofloxacin. The *in vivo* results clearly mirror the *in vitro* data (Fig. 2). Without gallstones, there is a significant decrease in CFU numbers after ciprofloxacin treatment (P = 0.0035) although not as dramatic as in the *in vitro* experiment (Fig. 3b). However, when growing in biofilms on gallstones, CFU numbers stay constant after 10 days of antibiotic treatment. These results are consistent with clinical reports that indicate an elevated level of antibiotic resistance in chronic carriers^7,35^, especially if they suffer from gallstones^9,19,21^. Notably, our results highlight the importance of the chronic carriage mouse model, as they recapitulate one of the hallmark characteristics of chronic Typhoid carriage: the correlation between the presence of gallstones and recalcitrance towards antibiotics, in this case ciprofloxacin.

Interestingly, there is a marked difference between the CFU numbers of the two untreated control groups, where mice on a normal diet showed a 2-logs higher CFUs than mice on a lithogenic diet. This differs from other observations previously made by our group, where gallbladders with gallstones usually present higher bacterial loads. A possible explanation might be the infection dose; in this experiment, 5 × 10^4^ CFUs/mL were injected IP as opposed to the 1 × 10^4^ CFUs/mL used in previous studies^36^.

Ciprofloxacin has been shown to effectively penetrate biofilms composed of different bacteria^29^ and works by inhibiting cell division by targeting DNA gyrase and topoisomerase IV^37^. This antibiotic is also more effective than other antibiotics *in vivo* against a wide range of Gram positive and negative bacteria^38^ and has excellent penetration inside tissue^39^. Additionally, it has been shown to significantly reduce *Salmonella* CFU in C57BL/6 mice, although some bacteria can survive in dendritic cells in some organs^40^. There are various hypotheses as to why biofilms are more resistant to antibiotics (including ciprofloxacin) than planktonic cells. These include slow or incomplete penetration of the antibiotic into the biofilm, an altered chemical microenvironment within the biofilm, and that a subpopulation of micro-organisms in a biofilm might be in a highly protected spore-like state (persisters)^41,42^. We have observed that *in vitro Salmonella* biofilms reach maximum CFU levels after about 48 hours and, even though the biofilm matrix increases in size, CFU numbers stay constant (data not shown), suggesting low levels of cell division, or persister cells, which could be protected from ciprofloxacin action.

Fluoroquinolone resistant isolates emerged soon after the widespread use of these antimicrobials and are now endemic in large parts of Asia and Africa. Such strains are also increasing in non-endemic areas like Europe and North America, primarily due to international travel^23^. A recent global phylogeographical analysis identified mutations in the primary targets of fluourquinolones, DNA gyrase subunits *gyrA* and *gyrB* and topoisomerase IV components *parC* and *parE*, more commonly in the MDR strain H58 than in other Typhi isolates^43^. Moreover, we have previously established a positive correlation between biofilm formation and antibiotic resistance, which could indicate that the biofilm state might provide an ideal environment for the transfer of resistance genes^44^. This is a worrying scenario as genetic resistance coupled with the inherent resistance of biofilms could render the elimination of carriers an arduous task. *S*. Typhi is a human-restricted pathogen and as such, humans are the only reservoir that spread the disease. As sanitation and access to clean water improves in developing countries, chronic carriers should play an even more important role in disease transmission^45,46^. Additionally, understanding the unique conditions of the gallbladder that allow *Salmonella* to establish a chronic infection will be a crucial step towards eliminating the disease.

## Methods

### Ethics statement

Mouse care and housing was carried out in accordance with guidelines established by the Ohio State University (OSU) Institutional Animal Care and Use Committee (IACUC). Animal work was previously approved by OSU IACUC. The Ohio State University Animal Care and Use Program is accredited by the Association for the Assessment and Accreditation of Laboratory Animal Care International (AAALAC). Research activities conformed to the statutes of the Animal Welfare Act and guidelines of the Public Health Service as required in the *Guide for the Care and Use of Laboratory Animals*.

### Bacterial Strains and Growth Conditions

*S*. Typhimurium strain 14028 (JSG210) and *S*. Typhi Ty2 (JSG624) were streaked on Luria-Bertani (LB) agar plates and incubated at 37 °C overnight. Single colonies were used to start overnight (O/N) liquid cultures. Planktonic cells were grown at 37 °C on a rotating drum in LB, or tryptic soy broth (TSB). Green fluorescent protein (GFP; pFPV25.1) expressing strains JSG1149 (*S*. Typhimurium) and JSG1150 (*S*. Typhi) were grown in Amp (100 μg ml^−1^) to keep selective pressure on the plasmid^13^.

### Biofilm growth

*S*. Typhimurium biofilms were grown on non-treated polystyrene 96-well plates (Corning, Kennebunkport, ME) by normalizing overnight (O/N) cultures grown in TSB to OD_600_ = 0.8, then diluting 1:10 into biofilm-growth media (TSB diluted 1:20), and dispensing 0.1 ml per well. The plates were incubated at 30 °C in a GyroMini nutating mixer (LabNet International, Inc., NJ) at 24 rpm. *S*. Typhi biofilms were grown as follows: O/N liquid cultures were incubated in TSB at 37 °C with aeration. These were then normalized to OD_490_ = 0.65, diluted 1:2500 in TSB, and 200 µL/well were dispensed into 8-well Permanox chamber slides (Thermo Fisher, Rochester NY) and incubated at 37 °C with 5% CO_2_. To simulate growth conditions on gallstones, wells were pre-coated with cholesterol by adding a solution of 5 mg/mL in 1:1 isopropanol:ethanol and air-dryed overnight^17^. Media was changed daily for both *S*. Typhimurium and *S*. Typhi biofilm growth.

### Antimicrobial Susceptibility Testing

Ciprofloxacin (≥98%, HPLC) was purchased from Sigma-Aldrich (St. Louis, MO). The Minimum Inhibitory Concentration (MIC_50_) of ciprofloxacin for planktonic *Salmonella* serovars was determined by the broth-dilution method^47^ using the CLSI guidelines for *Salmonella* ^15^. Biofilm MIC assays were performed in 96-well plates. Planktonic and biofilm bacteria were incubated (0–8 hours) in the presence or absence (control) of ciprofloxacin at or near the calculated MIC_50_ (0.125 μg/mL for *S*. Typhimurium and 0.02 μg/mL for *S*. Typhi). In order to evaluate antimicrobial susceptibility on an equal amount of cells, planktonic cultures were normalized to the biofilm cell concentration by CFU enumeration of a dilution series (5-day biofilm CFUs were previously determined, and planktonic CFUs were diluted to match this) and by total protein measurements using the bicinchoninic acid (BCA) method (Pierce/ThermoFisher). CFU numbers were calculated prior to antibiotic treatment (T_0_) then every 2 hours for a total of 8 hours. To detach biofilms from the abiotic surfaces, media was removed to discard planktonic cells. The matrix was then scratched from the surface of the plates with a 200 μL pipette tip and washed with 100 μL PBS twice. PBS was then added to a volume of 1 mL and vortexed on high speed for 60 to 75 s to disrupt cell aggregates. Serial dilutions were plated on LB agar.

### Murine Model of Typhoid Carriage

A total of 48 129X/SvJ (The Jackson Laboratory, Bar Harbor, ME) mice were used in this experiment. Half of the mice were fed a normal diet (ND) and the remaining half were fed a lithogenic diet (LD) for 8 weeks^12^. Mice from both ND and LD groups were injected intraperitoneally with a dose of 5 × 10^4^ CFUs/mL of *S*. Typhimurium or PBS (control). Five days post-infection, ciprofloxacin injections of 1 mg/kg/day were administered to half of the treatment and control mice daily for a 7-day period (Fig. 3a). At the end of the study, all mice were sacrificed and their gallbladders removed. Gallbladders were macerated with a TissueLyser LT (Qiagen, Valencia, Ca). Total bacterial concentration (CFUs/gallbladder) was calculated by serially diluting gallbladder macerates on LB agar plates.

### Confocal Microscopy

Established biofilms of strains JSG1149 and JSG1150 were incubated with or without ciprofloxacin for 8 hours prior to imaging. Biofilms were washed two times in PBS and fixed in 2% paraformaldehyde (PFA, Affimetrix, Cleveland, OH) for 20 minutes at room temperature at each time point and saved for later imaging. The amount of biofilm and the structure of the biofilm can be inferred by the amount of GFP signal detected^48^. Biomass and average thickness were assessed by automated capturing of 10 random Z-stacks per well in 5 wells per treatment using a Nikon A1R Live Cell Inverted Confocal microscope. The Z-stacks were then analyzed using the software package COMSTAT2^49^.

### Data Analysis

Planktonic and biofilm experiments were performed using 5 biological replicates and were repeated at least three times. CFU numbers were Log-transformed prior to analysis and statistical significance testing and was performed using R 3.2.3^50^ or Graph Prism 7. All p values < 0.05 were considered significant.
